# RNA Aptamers Generated against Oligomeric Aβ40 Recognize Common Amyloid Aptatopes with Low Specificity but High Sensitivity

**DOI:** 10.1371/journal.pone.0007694

**Published:** 2009-11-10

**Authors:** Farid Rahimi, Kazuma Murakami, Jamie L. Summers, Chi-Hong B. Chen, Gal Bitan

**Affiliations:** 1 Department of Neurology, David Geffen School of Medicine, University of California Los Angeles, Los Angeles, California, United States of America; 2 Department of Biological Chemistry, University of California Los Angeles, Los Angeles, California, United States of America; 3 Brain Research Institute, University of California Los Angeles, Los Angeles, California, United States of America; 4 Molecular Biology Institute, University of California Los Angeles, Los Angeles, California, United States of America; Mental Health Research Institute of Victoria, Australia

## Abstract

Aptamers are useful molecular recognition tools in research, diagnostics, and therapy. Despite promising results in other fields, aptamer use has remained scarce in amyloid research, including Alzheimer's disease (AD). AD is a progressive neurodegenerative disease believed to be caused by neurotoxic amyloid β-protein (Aβ) oligomers. Aβ oligomers therefore are an attractive target for development of diagnostic and therapeutic reagents. We used covalently-stabilized oligomers of the 40-residue form of Aβ (Aβ40) for aptamer selection. Despite gradually increasing the stringency of selection conditions, the selected aptamers did not recognize Aβ40 oligomers but reacted with fibrils of Aβ40, Aβ42, and several other amyloidogenic proteins. Aptamer reactivity with amyloid fibrils showed some degree of protein-sequence dependency. Significant fibril binding also was found for the naïve library and could not be eliminated by counter-selection using Aβ40 fibrils, suggesting that aptamer binding to amyloid fibrils was RNA-sequence-independent. Aptamer binding depended on fibrillogenesis and showed a lag phase. Interestingly, aptamers detected fibril formation with ≥15-fold higher sensitivity than thioflavin T (ThT), revealing substantial β-sheet and fibril formation undetected by ThT. The data suggest that under physiologic conditions, aptamers for oligomeric forms of amyloidogenic proteins cannot be selected due to high, non-specific affinity of oligonucleotides for amyloid fibrils. Nevertheless, the high sensitivity, whereby aptamers detect β-sheet formation, suggests that they can serve as superior amyloid recognition tools.

## Introduction

Alzheimer's disease (AD) is a progressive neurodegenerative disorder that initially presents as episodic memory lapses and culminates in the decline of mental faculties, dementia, and death. According to the Alzheimer's Association, an estimated 5.3 million Americans have AD in 2009 and this number will increase to 11–16 million by 2050 [1]. The annual cost of care for AD in the US is more than $ 148 billion and will increase dramatically if effective prevention and/or cure are not found. AD affliction typically occurs in the eighth or ninth decade of life with incidence rising steeply after age 65. Presymptomatic diagnosis of AD is difficult and clinical diagnosis relies on patients' history, cognitive assessment, and neuroimaging providing an overall sensitivity of ∼85% [2]. Definite diagnosis of AD is achieved only by postmortem neuropathological examination. Thus, an urgent need exists for development of diagnostics and early-intervention tools for AD. Because the onset of AD occurs insidiously many years before emergence of the initial symptoms [3], early diagnosis is particularly important for effective therapeutic intervention before progression to symptomatic disease [4].

Amyloid β-protein (Aβ) is central to the pathogenesis of AD. Presently, it is believed that soluble oligomeric, rather than fibrillar, Aβ assemblies act as the proximate neurotoxins that cause synaptic dysfunction and neuron loss in AD [5], [6]. Various forms of soluble Aβ assemblies, including low-molecular-weight (LMW) oligomers, Aβ-derived diffusible ligands, paranuclei, and protofibrils have been described [7]. However, the interrelationships amongst these Aβ oligomers and their relevance to AD etiology and pathogenesis remain unclear.

Biomarker discovery and specific molecular recognition tools may unravel the interrelationships amongst Aβ assemblies and facilitate detection and characterization of these assemblies early in the course of AD. Commonly, molecular recognition depends on antibodies, which are indispensable diagnostic tools in research and clinics. Many antibodies against Aβ have been developed, characterized, and used to enable highly specific detection of different Aβ assemblies [8]–[11]. Certain antibodies have been reported to recognize conformational epitopes in Aβ assemblies and react with similar assemblies of other amyloidogenic proteins [12]–[15], and some raised against oligomers recognize both oligomeric and fibrillar assemblies [15]–[17]. In the past two decades, an alternative class of molecular recognition tools, aptamers, has emerged offering important advantages relative to antibodies, including rapid selection *in vitro*, high stability under non-physiologic conditions, straightforward chemical manipulation, low immunogenicity, and cost-effectiveness [18], [19]. Particularly, single-stranded DNA (ssDNA) or RNA befit combinatorial selection approaches because they fold into well-defined three-dimensional structures and are easily amplified by polymerase chain reaction (PCR).

Despite the emergence of aptamers as tools in modern biotechnology and medicine [20], they have been underutilized in the amyloid field. Most of the RNA or ssDNA aptamers for amyloidogenic proteins have been selected against various forms of prion proteins (PrP) [21]–[25]. Bunka *et al*. have generated aptamers using monomeric and several forms of fibrillar β_2_-microglobulin (β_2_m). The aptamers were found to bind fibrils of certain other amyloidogenic proteins in addition to those of β_2_m [26]. In Aβ research, a single study described RNA aptamers selected against immobilized monomeric Aβ40 [27]. Despite selection against monomeric Aβ40, these aptamers showed binding to fibrillar Aβ40. These data raised several questions. Why did aptamers selected against monomeric proteins recognize their polymeric forms? Why did these aptamers not recognize the original targets, i.e., the monomeric forms of those proteins? Could aptamers against monomeric and/or oligomeric forms of amyloidogenic proteins be obtained?

To address these questions, here, we attempted to generate aptamers against covalently-stabilized oligomeric Aβ40 generated using photo-induced cross-linking of unmodified proteins (PICUP) [28]. As our first target, we chose a cross-linked Aβ40 trimer because it is the predominant product when Aβ40 is subjected to PICUP. We describe the aptamers obtained and discuss the implications and challenges of using *in vitro* selection of aptamers for fibrillar and non-fibrillar assemblies of amyloid proteins.

## Results

### Systematic Evolution of Ligands by EXponential Enrichment (SELEX) Targeting Covalently-Stabilized, Trimeric Aβ40

**Figure 1:**
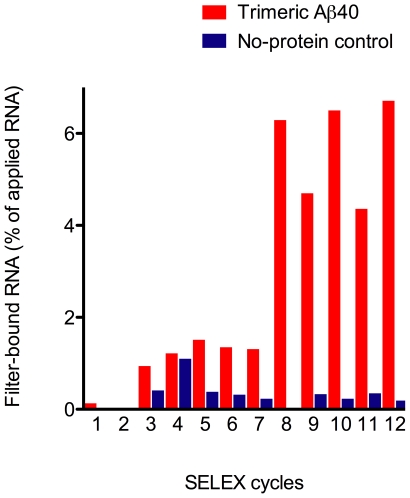
Progression of SELEX monitored by filter-binding assay. Amount of bound radioactivity for each round of selection was plotted as a percentage of total RNA count used for the corresponding cycle.

To target Aβ40 trimers by *in vitro* selection, covalently-stabilized Aβ40 oligomers were generated using PICUP [29] and trimers were purified using a newly developed protocol that allows high protein recovery following SDS-PAGE separation (Rosensweig C. J. *et al*., manuscript in preparation). We performed SELEX with the purified, cross-linked Aβ40 trimers using filter binding, and increased the stringency of the selection gradually during the process (see Materials and Methods). Progression of selection for each RNA pool was monitored by filter-binding assay and scintillation counting (Fig. 1). We observed 6.7% enrichment of the initial RNA pool after 12 cycles (Fig. 1). We then cloned the resulting aptamers and obtained 86 clones yielding 48 unique sequences, named KM1–KM48. Motif analysis of 38 sequences showed that all the aptamers had high (∼70%) G content and contained a conserved GGXGG motif.

**Figure 2:**
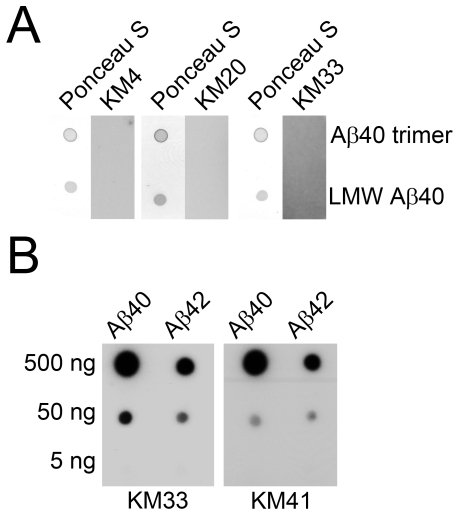
Characterization of aptamers selected against trimeric Aβ40. (A) LMW and trimeric Aβ40 were spotted at 100 pmol and membranes were probed with radioactively labeled RNA aptamers, KM4, KM20, or KM33. Blots were stained with Ponceau S after probing and washing to confirm protein retention. (B) Aβ40 or Aβ42 fibrils were sonicated for 10 min, spotted onto nitrocellulose membranes, and probed with aptamers KM33 and KM41.

In initial dot-blot screening experiments, we evaluated 33 unique clones for their reactivity with trimeric or LMW Aβ40 [30]. Dot blotting does not affect protein conformation during the time-course of these experiments [12], [31], [32]. Despite using stringent selection conditions, none of the aptamers reacted with trimeric or LMW Aβ40 (up to 400 ng (∼100 pmol) per spot). Low-molecular-weight proteins, such as Aβ, may not adsorb strongly to membranes and get desorbed during wash steps. Therefore, we confirmed protein adsorption and retention on blotting membranes by Ponceau S staining after incubation with aptamers and subsequent washes. Fig. 2A shows dot blotting and Ponceau S staining of trimeric and LMW Aβ40 for 3 representative aptamers—KM4, KM20, and KM33. Because aptamers selected against monomeric Aβ40 [27] or monomeric β_2_m [26] were reported to bind Aβ40 fibrils or various forms of β_2_m fibrils, respectively, we assessed the reactivity of KM aptamers against Aβ40 and Aβ42 fibrils. Similar to the previous observations [26], [27] and despite selection for covalently- stabilized trimeric Aβ40, all 33 unique aptamers bound Aβ40 and Aβ42 fibrils. KM33 and KM41 were selected out of the 33 clones for further characterization based on their apparent preferential binding to Aβ40 fibrils observed in our initial screening. Both KM33 and KM41 were found to react with as low as 50 ng (∼12 pmol) Aβ40 or Aβ42 fibrils (Fig. 2B).

### KM Aptamers Bind Other Amyloid Fibrils

**Figure 3:**
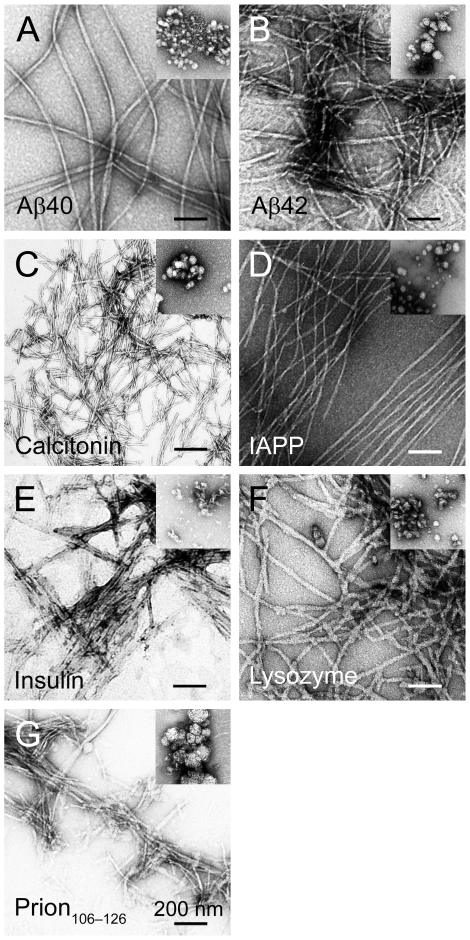
Morphologic characterization of fibrillar and unaggregated protein preparations. Electron micrographs of fibrillar Aβ40 (A), Aβ42 (B), calcitonin (C), IAPP (D), insulin (E), lysozyme (F), and prion_106–126_ (G). Insets show unaggregated preparations of the corresponding proteins. Scale bars represent 200 nm.

**Figure 4:**
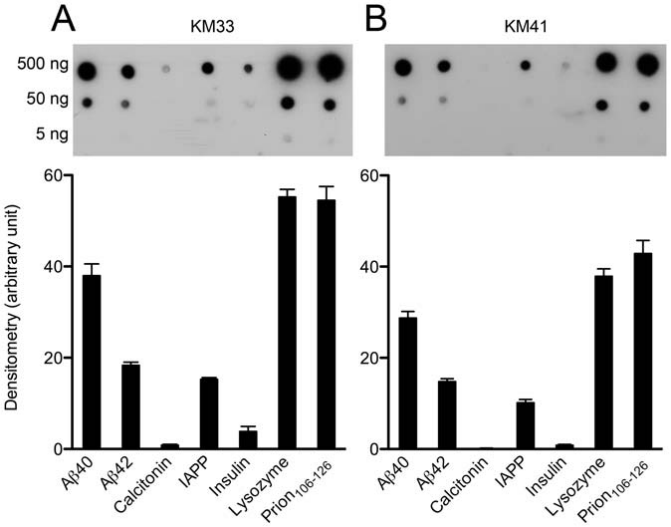
Reactivity of KM aptamers with different amyloid fibrils. Aβ40, Aβ42, calcitonin, IAPP, insulin, lysozyme, and prion_106–126_ were spotted (1 µL) onto GSWP nitrocellulose membranes and probed with KM33 (A) or KM41 (B). Each blot is representative of four independent experiments. Histograms show average densitometry values of the 500-ng protein spots. Values are expressed as mean±SEM.

Under appropriate conditions, proteins with unrelated primary structures form amyloid fibrils. The fibrils of these proteins share a common cross-β structure and characteristic tinctorial and morphologic properties [33]. Because certain antibodies have been shown to react with fibrillar forms of various amyloid proteins [13], [14], it is thought that this common cross-β structure in amyloid fibrils is the target. Thus, we assessed the selectivity of our aptamers for Aβ fibrils by examining whether they recognize fibrils of other amyloidogenic proteins, including calcitonin, islet amyloid polypeptide (IAPP), insulin, lysozyme, and prion_106–126_. Each protein was incubated under fibril-inducing conditions and fibrillar morphology was confirmed by electron microscopy (EM) (Fig. 3) prior to dot blotting. To compare aptamer reactivity quantitatively, we analyzed the intensities of the fibril spots containing the highest protein amount (500 ng) densitometrically. The reactivity of KM33 and KM41 with Aβ40 was significantly (*P*<0.001) stronger than with Aβ42 (Fig. 4, Table 1). Both KM33 and KM41 reacted with fibrils of other amyloid proteins. The aptamers reacted most strongly with lysozyme and prion_106–126_, whereas the reaction with calcitonin or insulin was relatively weak (Fig. 4, Table 1). The reactivity with lysozyme and prion_106–126_ was significantly different than with all the other protein fibrils (*P*<0.001). KM33 was more reactive with all protein fibrils compared to KM41 but this difference was statistically significant only for calcitonin (*P*<0.01).

**Table 1:** Densitometry values of the reactivity of aptamers with fibrillar amyloid proteins.

	KM33	KM41	β19‡	β37‡	β55‡	β61‡
Aβ40	38±2.5	28.8±1.4	22.3±4.5^**^	26.9±0.5*	22±1.5^**^	22.6±1^**^
Aβ42	18.4±0.6_•••_	14.9±0.6_•••_	10.5±1.5^***^	14.7±1_••_	10.5±0.6^***^	9.6±0.4^***^_••_
Calcitonin	0.9±0.1_•••_	0.1±0.06^**^_•••_	0.1±0.02^**^_••_	0.8±0.2_•••_	0.2±0.05^**^_••_	0.3±0.2*_•••_
IAPP	15.3±0.3_•••_	10.2±0.7_•••_	11.6±0.8	11.6±1.4_•••_	12.3±2.4	10.2±1.1_••_
Insulin	3.9±1_•••_	0.9±0.1_•••_	3.7±1.5_•_	4.5±1.6_•••_	2.8±0.9_••_	2.6±1.2_•••_
Lysozyme	55.3±1.6_•••_	37.9±1.6_••_	50±6.5_•••_	45.6±3.1_•••_	49.8±6.9_•••_	39.3±3.9_•••_
Prion_106–126_	54.5±3_•••_	42.9±2.8_•••_	48.2±4_•••_	48.7±3.8_•••_	47.2±3.6_•••_	42.7±3.6_•••_

* *P*≤0.05, ^**^ *P*≤0.01, and ^***^ *P*≤0.001, aptamer reactivity compared to KM33 reactivity with the same protein.

_•_ *P*≤0.05, _••_ *P*≤0.01, and _•••_ *P*≤0.001, reactivity of the same aptamer with Aβ40 compared with the reactivity with other proteins.

‡ Aptamers reported by Ylera *et al*. [27].

### Comparison of KM Aptamers with Previously Published Anti-Aβ40 Aptamers

**Figure 5:**
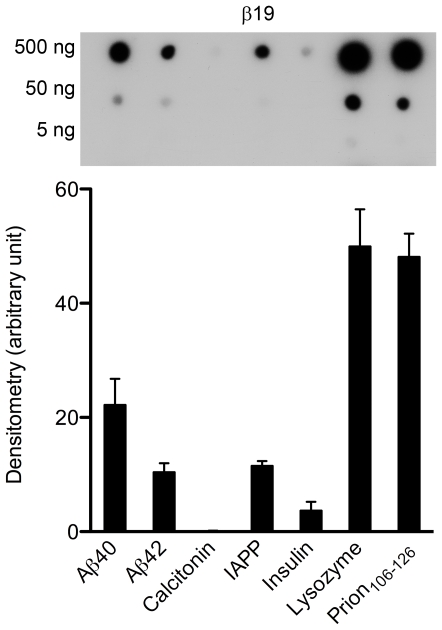
Reactivity of β19 aptamer with different amyloid fibrils. Aβ40, Aβ42, calcitonin, IAPP, insulin, lysozyme, and prion_106–126_ were spotted (1 µL) onto GSWP nitrocellulose membranes and probed with β19. The blot is representative of four independent experiments. Histograms show average densitometry values of the 500-ng protein spots. Values are expressed as mean±SEM.

To assess whether the reactivity of the KM aptamers with amyloid fibrils of different proteins was unique, we examined anti-Aβ40 aptamers published previously [27]. Those aptamers were selected against polymer-attached, *N*-terminally modified, Aβ40 monomers which were coupled to the polymer column in 60% 1,1,1,3,3,3-hexafluoro-2-propanol (HFIP), a solvent that promotes α-helix conformation and disaggregation of Aβ [34]. One aptamer, called β55, was shown by immunogold labeling and EM to bind Aβ40 fibrils [27]. We chose to examine four of the aptamers of Ylera *et al.* (Table 1) that were reported to have the highest affinity for polymer-attached Aβ40 determined by affinity chromatography. To test the selectivity of these aptamers, we used fibrillar preparations of Aβ and the 5 amyloidogenic proteins described above. The reactivity of all four aptamers with the proteins tested was similar and comparable to KM33 and KM41 (see Fig. 5 for an example and Table 1 for the entire data set). Similar to KM33 (Fig. 4A) and KM41 (Fig. 4B), the reactivity of the aptamers of Ylera *et al.* with Aβ42 fibrils was somewhat lower than their reactivity with Aβ40 fibrils, suggesting moderate preference for Aβ40.

### Counter-Selection Against Aβ40 Fibrils

**Figure 6:**
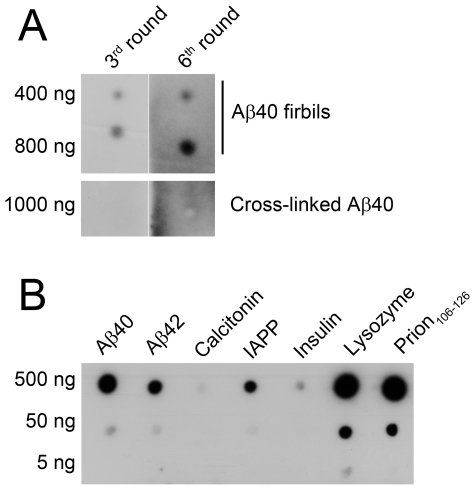
Reactivity of the 3^rd^ and the 6^th^ RNA pools with Aβ40 and other amyloid fibrils. (A) Six SELEX cycles were performed using cross-linked LMW Aβ40 and the progression of SELEX was assessed after two counter-selection cycles by testing the reactivity of the RNA pool for Aβ40 fibrils (400 and 800 ng) or cross-linked Aβ40 (1,000 ng). Blots were probed with RNA pools generated before (3^rd^ cycle) and after (6^th^ cycle) counter-selection. (B) Fibrillar preparations of Aβ40, Aβ42, calcitonin, IAPP, insulin, lysozyme, and prion_106–126_ were spotted at 5, 50, and 500 ng and the membranes were probed with the 6^th^ RNA pool.

**Figure 7:**
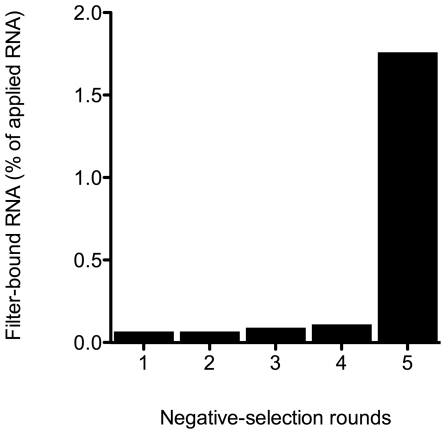
Progression of negative-selection rounds using Aβ40 fibrils. Amount of bound radioactivity for each round of negative selection was plotted as a percentage of total RNA count used for the corresponding cycle.

Because in both our study and previous attempts to select aptamers against monomeric forms of amyloid proteins [26], [27] the resultant aptamers were highly reactive towards amyloid fibrils and not the monomeric or oligomeric targets, in subsequent experiments, we attempted to counter-select against fibrils. In these experiments, we performed SELEX using a mixture of covalently-stabilized Aβ40 oligomers as target because this preparation was easier and faster to generate than gel-purified Aβ40 trimers. In addition, we suspected that if traces of SDS were present in the previous experiment, they might have accelerated Aβ self-assembly and β-sheet formation [35]. Using the oligomer mixture as a target alleviated the need for SDS-PAGE separation and avoided the presence of SDS in the preparation altogether. We performed 6 SELEX cycles with counter-selections against Aβ40 fibrils after the 4^th^ and 5^th^ cycles. We then assessed the 3^rd^ (before counter-selection) and the 6^th^ RNA pools for reactivity with the target—oligomeric Aβ40, and the unintended, cross-reactive species—Aβ40 fibrils. We found that both the 3^rd^ and the 6^th^ RNA pools reacted with Aβ40 fibrils but not with oligomeric Aβ40 indicating that the counter-selection against fibrils was inefficient (Fig. 6A). Although the mixed oligomers were covalently stabilized, their dynamic nature may cause relatively weak and/or short-lived RNA–peptide interactions during SELEX reducing the power of SELEX cycles to achieve selectivity. Moreover, the 6^th^ RNA pool showed the same pattern of reactivity with fibrils of Aβ and the other amyloidogenic proteins (Fig. 6B) as the KM aptamers (Fig. 4) and the aptamers generated by Ylera *et al*. (Fig. 5, Table 1). Because two counter-selections against Aβ40 fibrils failed to remove fibril reactivity of the 6^th^ RNA pool, we attempted to restart from the original RNA library and perform several “negative selections” against Aβ40 fibrils in an effort to obtain an RNA pool devoid of fibril-binding sequences. However, despite 5 consecutive rounds of negative selection against excess (2 µg) Aβ40 fibrils, the percentage of RNA binding to fibrils, rather than decreasing, increased from 0.1 to 1.7% (Fig. 7). Thus, similar to the counter-selection cycles, the negative selection experiments did not reduce RNA binding to Aβ40 fibrils.

### Assessment of Naïve Libraries

**Figure 8:**
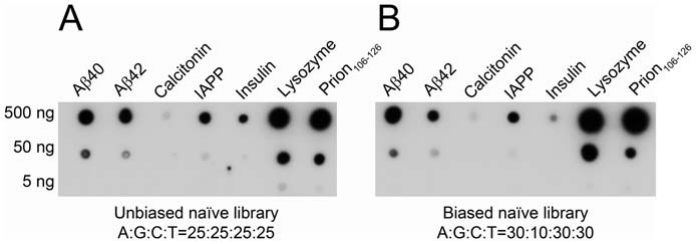
Naïve RNA libraries react with different amyloid fibrils in a pattern similar to individual aptamers. Fibrillar preparations of Aβ40, Aβ42, calcitonin, IAPP, insulin, lysozyme, and prion_106–126_ at 5, 50, and 500 ng/µL were sonicated for 10 min and 1 µL was spotted onto GSWP membranes. Membranes were probed with unbiased (A) or biased (B) library.

In view of the persistent and apparently non-specific binding of RNA aptamers to amyloid fibrils, we assessed whether RNA binding was found in the naïve library. In addition, because our sequencing and motif analyses showed high G content in KM aptamers, we also tested a biased library with reduced G ratio (A∶C∶G∶T = 30∶30∶10∶30%). We found that both libraries reacted with fibrils of Aβ40, Aβ42, and the other amyloid proteins (Fig. 8A, B) in a fashion similar to all the selected aptamers tested (Figs. 4 and 5).

### Aptamer Reactivity Depends on Protein Assembly State

Our experiments using the naïve libraries suggested that aptamer binding to fibrils reflected an inherent, sequence-independent affinity of ribo-oligonucleotides for the fibrillar structure of proteins. HFIP treatment has been reported to break apart β-sheets, disrupt hydrophobic interactions, and promote α-helical secondary structures [34]. Aβ40 and Aβ42 adopt predominantly α-helical conformation when treated with 100% HFIP [34]. This method has become standard in recent years for preparation of aggregate-free Aβ. Typically, the HFIP is evaporated to dryness resulting in a peptide film that can then be re-dissolved in the desired aqueous solution. Such “unaggregated” preparations of Aβ40 and Aβ42 have been obtained by dissolution of dry, HFIP-treated peptide films in dimethyl sulfoxide followed by dilution in phosphate-buffered saline [34]. These preparations have been shown previously to be fibril-free [34]. To assess RNA-sequence-independent affinity of aptamers for the protein assembly state, we tested the reactivity of single aptamers and the naïve library with HFIP-treated proteins both directly, and following “unaggregated” protein preparations.

**Figure 9:**
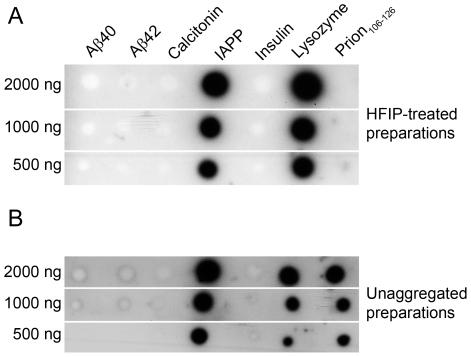
Reactivity of KM33 with HFIP-treated and “unaggregated” proteins. HFIP-treated [34] (A) or unaggregated (B) preparations of Aβ40, Aβ42, calcitonin, IAPP, insulin, lysozyme, and prion_106–126_ were spotted at 500–2000 ng and probed with KM33.

Prior to blotting, we examined the morphology of “unaggregated” proteins as shown in Fig. 3. An example of a dot blot experiment is shown for KM33 in Fig. 9. KM33 did not react with HFIP-treated Aβ40, Aβ42, calcitonin, insulin, or prion_106–126_. However, it recognized HFIP-treated IAPP and lysozyme (Fig. 9A), demonstrating that HFIP treatment of the latter two proteins did not remove all the structural elements targeted by KM33. Similarly, unaggregated preparations of Aβ40, Aβ42, calcitonin, and insulin were not recognized by KM33 (Fig. 9B). Unaggregated IAPP, lysozyme, and prion_106–126_ reacted with KM33 strongly suggesting that they contained sufficient structural elements to be detected by KM33 (Fig. 9B). Similar patterns of reactivity were observed with KM41, all four of Ylera's aptamers, and the naïve library (data not shown).

### Evaluation of Aptamer Sensitivity

Aptamer reactivity with unaggregated and HFIP-treated IAPP, and lysozyme suggested that even following HFIP treatment, these proteins may contain sufficient β-sheet structure for aptamer recognition. The behavior of prion_106–126_ suggested that HFIP treatment disaggregated the peptide and disrupted β-sheet structure, but those were rapidly formed upon dissolution in buffer. Because β-sheet content increases with time during fibrillogenesis, we tested the sensitivity of aptamers for formation of the β-sheet-rich fibrillar structure in two proteins. KM41, β19, and the naïve library were used to probe the fibrillogenensis of Aβ40 and insulin in this experiment. The two proteins were chosen because their fibrillogenesis kinetics is relatively slow under the conditions we used and allow reliable measurement. We performed dot blotting simultaneously with thioflavin T (ThT) fluorescence assay and examination by EM. The ThT assay has been used commonly to study fibril formation in a number of amyloidogenic proteins [36]. ThT micelles bind to β-sheet structures resulting in an intensified fluorescence signal relative to unbound ThT [37].

**Figure 10:**
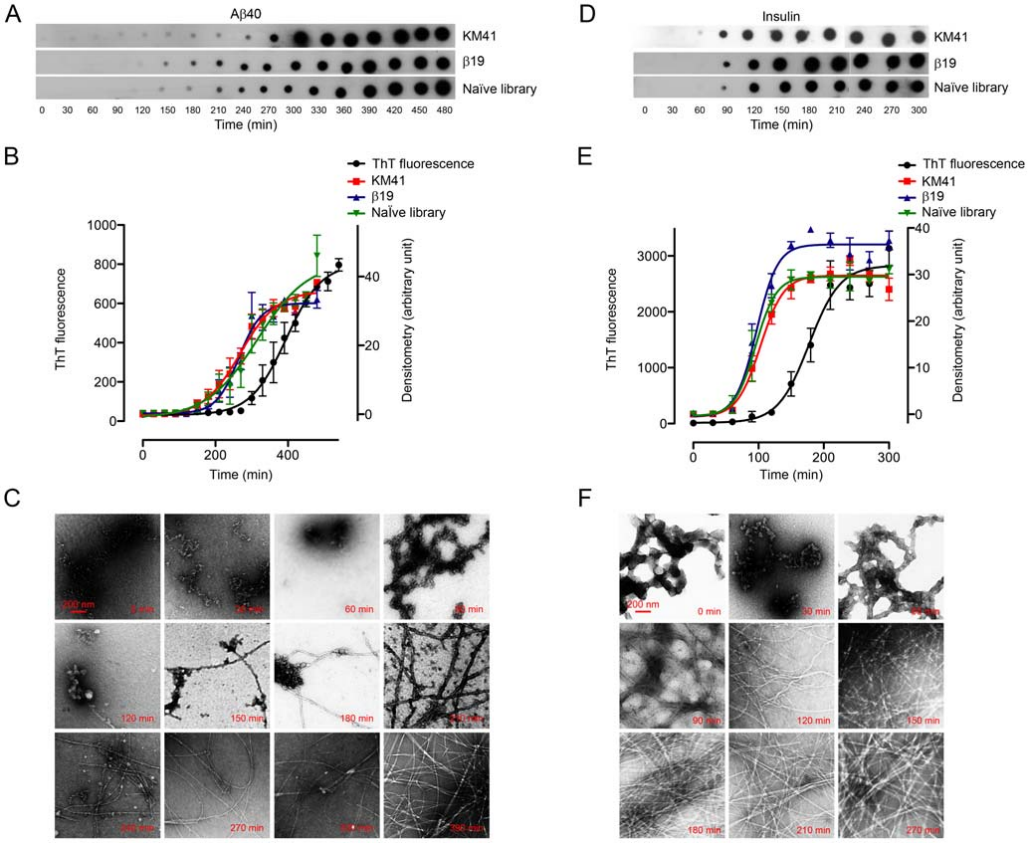
Comparison of ThT and aptamer detection sensitivity for β-sheet formation. Aβ40 (A–C) or insulin (D–F) were incubated under fibril-inducing conditions at a nominal concentration ∼1 mg/mL. One-µL aliquots of Aβ40 (A) or insulin (D) solution were spotted every 30 min for 480 or 300 min, respectively. The blots were probed with aptamers KM41 or β19, or the naïve library. Spot intensities were quantified densitometrically for 3 replicates, averaged, and plotted (B, E). Simultaneously, ThT fluorescence was recorded and compared with dot-blot densitometry data. Aliquots were examined in parallel by EM (C, F). The scale bar denoting 200 nm applies to all EM panels.

Initially, the aptamers or library did not bind to non-fibrillar Aβ40 or insulin (Fig. 10). Binding was observed after 90–120 min and increased gradually suggesting that aptamer binding correlated with the increase in β-sheet content. Both with Aβ40 and insulin, the aptamers recognized β-sheet conformation substantially earlier than ThT fluorescence even though the amount of protein in the dot-blot assay was 10-fold lower than in the ThT assay (Fig. 10). Simultaneous EM examination indicated that aptamer signals increased as soon as the first sparse fibrils formed in both Aβ40 (120 min; Fig. 10C) and insulin (60 min; Fig. 10F) solutions. In contrast, ThT fluorescence started increasing only when fibrillar species of Aβ40 (270 min; Fig. 10C) or insulin (120 min; Fig. 10F) were considerably more profuse. To compare the aptamer and ThT sensitivities quantitatively, we calculated the half-maximal time required to observe full reactivity (T*_50_*). When assessing the time-course of Aβ40 fibril formation, the T*_50_* values for KM41, β19, and the naïve library (Fig. 10B; Table 2) were significantly shorter (*P*<0.01) than that of the ThT fluorescence assay (Fig. 10B; Table 2). Similarly, T*_50_* values for KM41, β19, and the naïve library for insulin fibril formation were significantly shorter (*P*<0.01) than that of the ThT fluorescence assay (Fig. 10E; Table 2). Because 10-times more protein (∼10 µg) was used for each time point in the ThT assay compared to dot blotting (1 µg), these results demonstrate that aptamers detect the increase in β-sheet content with 15–17-fold higher sensitivity compared to ThT fluoresence.

**Table 2:** Half-maximal T values (T_50_±SE min) of aptamers and ThT fluorescence assay for β-sheet detection.

	Aβ40	Insulin
KM41	268±8**	103±4**
β19	264±7**	97±4**
Naïve library	315±20**	95±4**
ThT fluorescence	395±9	178±7

** *P*≤0.01 compared to ThT fluorescence assay.

## Discussion

Despite FDA approval of the first aptamer-based medication in 2005 [38], aptamers have been applied rarely to the amyloid field. Several groups reported development of aptamers against prion sequences [22]–[24], [39], and aptamer-based detection assays for prion proteins are now being developed [40]. Recently, aptamers generated against various fibrillar forms of β_2_m were reported [26]. An aptamer designated WL-2, generated against worm-like β_2_m fibrils, was found to recognize three different fibrillar forms of β_2_m and did not react with fibrils of apomyoglobin, Aβ40, or transthyretin (TTR). Remarkably, however, this aptamer bound strongly to lysozyme fibrils [26]. Based on the data presented here, this difference in reactivity likely reflects the higher non-specific affinity of RNA oligonuclotides for lysozyme than for Aβ40 (and presumably also apomyoglobin and TTR, which we did not use). Another aptamer selected against monomeric β_2_m, was shown to bind long, straight β_2_m fibrils [26]. An RNA aptamer generated against recombinant bovine PrP was shown to recognize bovine PrP-β [41], a soluble, oligomeric, β-sheet-rich, conformational variant of full-length PrP that forms amyloid fibrils [42].

Ylera *et al*. [27] selected aptamers against Aβ40 coupled to a Sepharose column through an engineered *N*-terminal cysteine. The protein was coupled to the column under α-helix–promoting conditions and the selected aptamers bound to the Sepharose-coupled Aβ40 with dissociation constants of 29–48 nM. However, aptamer binding to Aβ40 fibrils only was found and counter-elution with soluble Aβ40 did not lead to release of the aptamers from the support column [27]. The authors suggested that the cross-reactivity of the aptamers with Aβ40 fibrils was due to potential aggregation of Aβ40 in the support column [27] despite the use of 60% HFIP during the protein-coupling step. However, this explanation is unlikely in light of data reported by Fezoui *et al*. [43] showing that in a 60% 2,2,2-trifluoroethanol (TFE) solution, Aβ40 is predominantly α-helical and non-aggregated. TFE is a weaker inducer of α-helical conformation in Aβ than HFIP [43]. Thus, efforts to generate aptamers for amyloidogenic proteins have yielded sequences recognizing fibrillar assemblies of these proteins but not their monomeric form. Attempts to generate aptamers that would recognize specifically oligomeric forms also have been unsuccessful likely due to the dynamic nature of the oligomers preventing long-lasting RNA–oligomer interactions and their capture, perpetuation, and enrichment during SELEX cycles.

In an effort to generate aptamers selective for oligomeric Aβ and understand the reasons for the difficulty in achieving this goal, we targeted covalently-stabilized, PICUP-generated trimers of Aβ40. Despite selection for 12 cycles with increasing stringency, the selected aptamers did not react with trimeric or LMW Aβ40 but bound Aβ fibrils (Figs. 2, 4, 10), similar to the studies mentioned above [26], [27], [41]. Thus, we were unsuccessful in generating oligomer-specific aptamers. However, we gained significant insight into the reasons for this failure. Fibril reactivity of our aptamers was not unique to Aβ as they also recognized the fibrillar assemblies of five other amyloidogenic proteins (Fig. 4, Table 1) with different affinities. The data obtained by us and others suggest that aptamers generated against amyloidogenic proteins recognize a structural motif that is common to the fibrillar form of these proteins. This motif, likely is the backbone of the proteins in a cross-β structure. The reactivity clearly is RNA sequence-independent but depends on the protein assembly state and to some extent on the protein sequence.

The inherent and persistent tendency of amyloid fibrils to bind RNA may explain the observation that senile plaques and neurofibrillary tangles, the two pathological hallmarks of AD brains, contain RNA [44]–[46]. It has been suggested that amyloid fibrils and oligonucleotides may act as polyelectrolytes interacting by electrostatic forces [47]. Such β-sheet-mediated protein–nucleic-acid interactions may have been essential for scaffolding, stability, compartmentalization, protection, and degradation resistance under the harsh conditions of the primordial, prebiotic world [48].

Commensurate with the idea that electrostatic interactions are an important determinant of aptamer–fibril interaction, the preferential binding of the aptamers (and libraries) used here for lysozyme and prion_106–126_, correlate with the high pI values of these proteins, 9.82 and 10.00, respectively. However, isoelectric point (or net positive charge) alone do not account for the pattern of reactivity we observed because linear regression analysis of aptamer affinity *versus* pI yields weak correlation (*r^2^* = 0.42). The actual affinity likely depends on a number of factors, including the number of aptaopes per unit mass, exposure and availability of aptatopes under particular experimental conditions, and percent β-sheet content, in addition to the fibril surface charge.

Our data demonstrate that incubation in HFIP, a common treatment which presumably provides aggregate-free starting material for biophysical and biological studies of amyloidogenic proteins, is useful for Aβ40, Aβ42, calcitonin, and insulin but not entirely efficient for disaggregating IAPP and lysozyme, and its utility is questionable for prion_106–126_ (Fig. 9). Our data for Aβ are in agreement with those of Stine *et al*. who have reported that unaggregated and HFIP-treated preparations of Aβ40/Aβ42 were aggregate-free and contained <1% β-sheet measured by circular dichroism spectroscopy [34].

Owing to their inherent non-specific tendency to bind amyloid fibrils, oligonucleotides may be suitable as highly sensitive amyloid detectors. Our results indicate that aptamers can be used to detect early β-sheet formation more sensitively than the common ThT assay (Fig. 10) and that despite its popularity, the ThT assay does not detect β-sheet content formed in protein samples during early fibrillogenesis. Thus, these aptamers may facilitate highly efficient detection of advent of β-sheet formation in histopathological and in biophysical studies *in vitro*. Achieving high-sensitivity, aptamer-based detection of β-sheet formation depends on further development and streamlining of fast, refined, and easy quantitative assays. These assays potentially will complement methods that assess β-sheet-formation kinetics.

Aptamers have been shown to discriminate targets on the basis of subtle structural differences [49], [50]. Exploitation of such a selective power is yet to be achieved for aptamers in the amyloid field. If aptamers are to be obtained for diagnostic and therapeutic applications in amyloid diseases, future experiments aimed at generating avid and specific aptamers for pre-fibrillar assemblies, including monomers and oligomers, will have to address the inherent affinity of oligonucleotides for fibrillar structures.

## Materials and Methods

### Protein Preparation

Aβ40 was synthesized and characterized as reported previously [51]. For initial experiments, trimeric Aβ40 was used as the target for *in vitro* selection. Covalently-stabilized Aβ40 oligomers were generated using PICUP [28] and purified as described elsewhere (Rosensweig C. J. *et al*., manuscript in preparation). Briefly, following SDS-PAGE fractionation using 10–20% gradient Tricine gels (Invitrogen, Carlsbad, CA) and SimplyBlue Coomassie staining (Invitrogen), Aβ40 dimer (for counter-selection) and trimer (for selection) bands were excised off the gels. The gel bands were diced into small pieces, washed thrice in deionized water (18.2Ω, Millipore, Bedford, MA), subjected to 3 freeze–thaw cycles to make them brittle, and finally crushed by a mini-pestle. The crushed acrylamide was rotated end-over-end in 0.1% NH_4_OH for 1 h at 23°C to allow protein extraction. After centrifugation (750 *g*, 10 min, 23°C), the supernates were treated with SDS-OUT (Pierce, Rockford, IL) to remove SDS. Then, samples were dialyzed using 2,000-Da molecular weight cutoff Spectra/Por dialysis membranes (Spectrum Laboratories, Rancho Dominguez, CA) first against 10 M urea (4°C for 12 h), followed by ten changes of deionized water over 48 h to remove remaining SDS and Coomassie stain. The resultant solution was collected, lyophilized and kept at −80°C until use for aptamer selection.

In experiments using a mixture of PICUP-stabilized Aβ40 oligomers as the target for SELEX, cross-linked oligomers were subjected to buffer exchange and cross-linking reagents were removed using D-Salt Excellulose Desalting Columns (Thermo Fisher Scientific, Rockford, IL) in 10 mM ammonium acetate, pH 8.3. The cross-linked mixture of Aβ40 monomer and oligomers was lyophilized and treated with HFIP as described previously [52]. The HFIP then was evaporated and the protein films stored at −20°C. The integrity of the PICUP-generated oligomers was assessed by analyzing an aliquot using SDS-PAGE. Because SDS may induce artificial Aβ oligomerization [35], in separate experiments, cross-linked Aβ40 oligomer mixtures were analyzed by both SDS-PAGE and size-exclusion chromatography, which confirmed the presence of *bona fide* dimers, trimers, and tetramers (data not shown). Protein concentrations were determined using amino acid analysis.

### In Vitro Selection Using Aβ40 Trimers

A synthetic ssDNA library (Integrated DNA Technologies, Coralville, IA) for SELEX [53], [54], included 49 randomized nucleotides (A∶T∶G∶C = 25∶25∶25∶25%) flanked by constant regions that incorporated cloning sites (*Bam*HI, *Eco*RI) and a T7 promoter, as described previously [55] (Fig. 1). Using the double-stranded, PCR-amplified DNA template, RiboMAX Large-Scale RNA Production System-T7 (Promega, Madison, WI) was used to transcribe ^32^P-labeled RNA by internally incorporating α^32^P-cytidine triphosphate (GE Healthcare, Piscataway, NJ or Perkin Elmer, Waltham, MA). After phenol-chloroform extraction and desalting using Illustra MicroSpin G-50 columns (GE Healthcare), RNA integrity was confirmed by electrophoresis using 6% Tris-borate-EDTA-urea acrylamide gels (Invitrogen) and autoradiography for 30–60 min. RNA quantification was performed by scintillation counting using a Triathler bench-top scintillation counter (Hidex Oy, Turku, Finland).

Aptamers were selected in 12 rounds of SELEX using filter-binding for partitioning and assaying the bound RNA pool [55]. Before each round of SELEX, RNA was denatured at 90°C for 10 min and renatured at 23°C for 10 min. The RNA pool was pre-cleared using 0.45-µm pore size, HAWP02500 nitrocellulose filters (Millipore) to remove non-specific, filter-binding sequences before each selection round. Because Aβ40 trimers were purified off Coomassie-stained gels, RNA pools also were pre-cleared using Coomassie-stained HAWP filters after the 8^th^ and 12^th^ SELEX rounds to remove sequences that would potentially bind the Coomassie stain. Counter-selection against cross-linked dimeric Aβ40 was performed before the 5^th^ and 10^th^ SELEX rounds.

Renatured RNA pool (100 pmol, ∼6×10^13^ different sequences) and trimeric Aβ40 (400 pmol) were incubated in binding buffer (140 mM NaCl, 2.7 mM KCl, 10 mM Na_2_HPO_4_, 2 mM KH_2_PO_4_, pH 7.4) at 23°C for 60 min before separating the protein-bound RNA from the unbound fraction. Over the course of SELEX, the protein:RNA ratio was decreased gradually from 4∶1 to 1∶1 to 1∶2 in the first 7 rounds, next 3 rounds, and final 2 rounds, respectively. tRNA (10 µg/mL) was added to the selection reaction in the last 3 rounds to compete off non-specific RNA binding. After washing the filter 5 times with wash buffer (120 mM NaCl, 8 mM Na_2_HPO_4_, 1.9 mM NaH_2_PO_4_, 4.5 mM KCl, 0.12 mM MgSO_4_, 40 mM N-(2-hydroxyethyl)piperazine-N'-(2-ethanesulfonic acid), pH 7.4), the amount of filter-bound RNA was measured by scintillation counting of the filter and eluted in 7M urea, 100 mM sodium citrate, 3 mM EDTA, pH 7.0, as described previously [55]. Subsequently, the RNA pool was reverse-transcribed into cDNA by the ImProm-II Reverse Transcription System (Promega) and amplified by PCR followed by *in vitro* transcription for the next selection rounds. After the last cycle, individual aptamer clones were obtained by ligation of the PCR products into pGEM3Z vectors after digestion with *Eco*RI and *Bam*HI (USB, Cleveland, OH).

### In Vitro Selection Using Mixed PICUP-Stabilized Aβ40 Oligomers

Aβ40 oligomers were reconstituted in Tris-buffered saline (TBS, 10 mM Tris-HCl, 150 mM NaCl, 5 mM MgCl_2_, 1 mM EDTA, pH 7.5) at 1–3 µM. Because the aptamers selected for cross-linked Aβ40 trimers were found to bind fibrils (see Results), we were concerned that fibril formation by cross-linked oligomers might have occurred during incubation of oligomers with RNA. To assess whether that was the case, 3 µM of the mixture of cross-linked oligomers were incubated under identical conditions to the ones used for selection (in the absence of RNA) and monitored by turbidometry at A_400_ nm. Following a ∼45–60 min lag phase, the turbidity increased gradually and then reached a plateau, indicating particle growth. Based on these data, to ensure that no aggregation took place during incubation of RNA with the cross-linked oligomers, the incubation duration was shortened to 15 min. Three hundred to 1,000 pmol cross-linked Aβ40 oligomers were incubated with the RNA pool. The amount of RNA incubated with the protein target was reduced gradually from ∼100 nmol (∼6×10^16^ different sequences) in the first cycle to 4 nmol in the 6^th^ cycle. For filter binding in this case we used 0.22-µm pore-size nitrocellulose GSWP02500 membranes (Millipore) to minimize protein loss observed in the previous experiment using 0.45-µm pore-size membranes. Pre-clearing of the RNA pool against the GSWP filters was performed before each cycle starting at round 3. Two counter-selection rounds against 10 or 20 nmol Aβ40 fibrils were performed after the 4^th^ and 5^th^ SELEX cycles, respectively. The number of washes with TBS (0.5 mL each) was increased from 2 in the first 4 cycles to 6 in round 5, and to 10 in the 6^th^ cycle to increase selection stringency. tRNA (2.5 µg/µL) was incubated with the mixture of RNA and the target in the 6^th^ cycle for increased competition. After the 6^th^ cycle, the SELEX progression and selectivity were monitored by assessing the reactivity of the RNA pool with Aβ40 oligomers and fibrils.

### Anti-Aβ40 Aptamers Previously Published by Ylera et al

RNA sequences of aptamers designated β19, β37, β55, and β61 have been published previously ([27] and Dissertation by Ylera Dahmen, Francisco entitled “Selektion hochaffiner RNA-moleküle gegen das Alzheimer β-amyloid”, Freie Universität Berlin (1999) available at http://deposit.ddb.de/cgi-bin/dokserv?idn=961044268). Complementary DNA sequences for these aptamers and primers A (5′-TAATACGACTCACTATAGGGAATTCGA-GCTCGGTACC-3′) and B (5′-CCAAGCTTGCATGCCTGCAG-3′) were obtained from Integrated DNA Technologies. PCR was used to amplify the DNA sequences and to incorporate a T7 promoter using primer A. RNA aptamers were produced by *in vitro* transcription and ^32^P-labeled as described above.

### Aptamer Sequencing and Analysis of Secondary Structure

Cloned DNA was sequenced using an Applied Biosystems 3730 DNA Analyzer (Applied Biosystems, Foster city, CA) at the UCLA GenoSeq Genotyping and Sequencing Core Facility. Alignment search, classification, and local supermotif analysis were performed using sequences of 33 unique aptamers by Multiple Expectation maximization for Motif Elucidation [56] and Clustal W, version 2.0 [57]. To identify common secondary structural motifs, RNA sequences were folded using the energy minimization algorithm of Mfold [58].

### Preparation of Non-Fibrillar Protein Assemblies

LMW Aβ40 was prepared as described previously [30]. “Unaggregated” preparations of human Aβ40, Aβ42, calcitonin (American Peptide Company), IAPP (AnaSpec Inc., San Jose, CA), insulin (Sigma, St Louis, MO), lysozyme (Sigma), and prion_106–126_ (American Peptide Company, Sunnyvale, CA) were prepared as described previously [34]. Alternatively, HFIP-treated proteins were prepared as described previously [52]. Unaggregated and HFIP-treated proteins were used immediately for dot-blot analysis. Aliquots of unaggregated preparations were taken for EM analysis.

### Preparation of Fibrillar Protein Assemblies

All solvents were prepared under RNase-free conditions, contained 0.02% sodium azide to prevent bacterial growth, and were filtered through 0.02-µm Anotop filters (Whatman International Ltd. Maidstone, England) to remove particulate material. Aβ fibrils were prepared by reconstituting lyophilized peptides at 2 mg/mL in 6 mM NaOH in deionized water. Peptide solutions were sonicated for 2 min, then diluted with an equal volume of 20 mM phosphate, pH 7.4, and centrifuged at 16,000 *g* to precipitate large aggregates and obtain a homogeneous starting solution. Supernates were incubated at 37°C with rotary agitation (200 rpm) for 1 week. Calcitonin [59], IAPP [60], insulin [61], lysozyme [62], and prion_106–126_ [63] fibrils were prepared under appropriate conditions for each protein as described previously. Once fibrils formed, the preparations were washed 5 times in 10 mM phosphate buffer, pH 7.5, centrifuged after each wash to remove non-fibrillar material, quantified by amino acid analysis, and the fibrils finally suspended in buffer and stored at −20°C.

### Morphological Characterization of Protein Assemblies

The morphology of all fibrillar and unaggregated protein preparations was assessed by EM with negative staining. Briefly, 8–10 µL of protein preparation were applied to glow-discharged, carbon-coated Formvar grids (Electron Microscopy Sciences, Washington, PA) for 10–20 min. The solution was gently wicked off using Whatman grade-1 qualitative filter paper. Grids were treated with 10 µL of 0.5% (v/v) glutaraldehyde for 1 min and wicked dry. After rinsing with two 10-µL aliquots of water, samples were stained with 10 µL of 2% uranyl acetate (w/v, Pfaltz & Bauer, Inc., Waterbury, CT) for 2 min. The stain solution was wicked off and grids air-dried. Grids were examined using a Core JEOL Electron Microscope at 29,000× magnification at the UCLA Brain Research Institute Microscopy Core Facilities.

### Dot Blotting, Densitometry, and Statistics

All fibrillar preparations were sonicated for 10 min prior to dot blotting to produce fragmented short fibrils allowing uniform suspension, and diluted in a range of concentrations (5–500 ng/µL) before spotting. After mixing, 1-µL aliquots were spotted onto 0.2-µm pore-size, custom-ordered 9×6 cm, GSWP nitrocellulose membranes (Millipore) and air-dried. Non-fibrillar protein preparations were spotted in 1–2-µL aliquots to obtain the desired protein amount (500–2,000 ng) immediately after protein solubilization. Blots were rinsed and incubated in TBS. Each aptamer was denatured by pre-heating at 90°C (10 min), renatured by cooling to 23°C (10 min), and added to the buffer (5×10^5^ cpm/mL, 23°C). After 30 min, blots of fibrillar preparations were washed (10-min cycles, typically 7–10 times) in TBS containing 0.05% (v/v) Tween-20 (Sigma) until only background radioactivity was detectable in the wash buffer. TBS containing Tween-20 was found to desorb non-fibrillar preparations off the membrane following incubation with aptamers. Therefore, blots of unaggregated or HFIP-treated protein preparations were washed multiple times in TBS excluding Tween-20 until no radioactivity was found in the rinse. Blots were dried, wrapped in plastic wraps, exposed to X-ray films for 72 h, and developed. Subsequently, blots were stained with Ponceau S (3-hydroxy-4-(2-sulfo-4-[4-sulfophenylazo]phenylazo)-2,7-naphthalenedisulfonic acid; Amresco, Solon, OH) to confirm protein retention.

The fibril spots containing the highest protein amount (500 ng) were analyzed by densitometry using Gilles Carpentier's Dot-Blot-Analyzer macro (written by Gilles Carpentier, 2008. The macro is available at http://rsb.info.nih.gov/ij/macros/toolsets/Dot%20Blot%20Analyzer.txt and more information can be found at http://image.bio.methods.free.fr/dotblot.html) written for ImageJ [64]. Data are expressed as (mean±SEM). One-way analysis of variance (ANOVA) was performed and Tukey's test assessed differences which were considered significant if *P*≤0.05.

### Comparison of Thioflavin T Fluorescence Assay and Aptamer Detection Sensitivity

To assess progression of fibril formation, thioflavin T (ThT) assay, dot blotting, and EM were used to monitor solutions of Aβ40 or insulin contemporaneously. The reactions were performed in triplicates. ThT and dot-blot assays were used to monitor all 3 replicates whereas EM was used to monitor one out of the three. Three mM stock ThT solution prepared in deionized water was stored at 4°C in the dark to prevent photo bleaching. For insulin (nominally 1 mg/mL; prepared as above), 10-µL aliquots of the solution were added to 300 µL of 30 µM ThT in 50 mM glycine/NaOH, pH 8.0. For Aβ40 ThT assay, 10-µL aliquots of Aβ40 (nominally 1 mg/mL; prepared as above) were added to 300 µL of 30 µM ThT in 10 mM phosphate buffer, pH 7.5, because these conditions gave higher sensitivity than glycine/NaOH buffer. The fluorescence emission spectra were recorded following a 5-min incubation using an F-4500 Fluorescence Spectrophotometer (Hitachi High-Technologies Corporation, Japan) with λ_ex_ = 450 nm, 5-nm slit and λ_em_ = 482 nm, 10-nm slit. Disposable, Plastibrand, semi-micro UV cuvettes (Fisher Scientific, Pittsburgh, PA) with a 1-cm excitation light path were used for fluorescence spectrophotometry. EM grids were prepared as described above. For dot blots, 1 µL (∼1 µg protein) was spotted on GSWP membranes at each time point and subsequently probed with aptamers. Blots were incubated with aptamers as described above and developed by autoradiography. The films were scanned and the dot-blotting densitometry and ThT data were fitted using the Boltzmann sigmoidal function: 
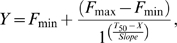
 where *F_max_* and *F_min_* denote the fluorescence/densitometry measures, respectively, and *T_50_* denotes the time when the fluorescence/densitometry values are half-maximal between *F_min_* and *F_max_*. T*_50_* values were calculated using Prism 5.0b (GraphPad Software, Inc., La Jolla, CA), expressed as average±SE, and statistical significance was calculated by ANOVA followed by Dunnett's Multiple Comparison test assessing pair-wise differences which were considered significant when *P*≤0.05.
